# Open semantic annotation of scientific publications using DOMEO

**DOI:** 10.1186/2041-1480-3-S1-S1

**Published:** 2012-04-24

**Authors:** Paolo Ciccarese, Marco Ocana, Tim Clark

**Affiliations:** 1 Harvard Medical School and Massachusetts General Hospital, Boston MA, USA; 2 Balboa Systems, Newton MA, USA; 3 University of Manchester, School of Computer Science, Manchester, UK

## Abstract

**Background:**

Our group has developed a useful shared software framework for performing, versioning, sharing and viewing Web annotations of a number of kinds, using an open representation model.

**Methods:**

The Domeo Annotation Tool was developed in tandem with this open model, the Annotation Ontology (AO). Development of both the Annotation Framework and the open model was driven by requirements of several different types of alpha users, including bench scientists and biomedical curators from university research labs, online scientific communities, publishing and pharmaceutical companies.

Several use cases were incrementally implemented by the toolkit. These use cases in biomedical communications include personal note-taking, group document annotation, semantic tagging, claim-evidence-context extraction, reagent tagging, and curation of textmining results from entity extraction algorithms.

**Results:**

We report on the Domeo user interface here. Domeo has been deployed in beta release as part of the NIH Neuroscience Information Framework (NIF, http://www.neuinfo.org) and is scheduled for production deployment in the NIF’s next full release.

Future papers will describe other aspects of this work in detail, including Annotation Framework Services and components for integrating with external textmining services, such as the NCBO Annotator web service, and with other textmining applications using the Apache UIMA framework.

## Background

Annotation is a fundamental activity in biomedical research and in scholarship generally. It associates a commentary or formal judgment (textual comment, revision, citation, classification, or other related object) to a target of annotation, such as a text or image. It can be created for personal use, as in note-taking and personal classification of documents. Or it can be addressed to an audience beyond its creator, as in shared commentary on documents, reviewing, citation, and tagging.

On the Web, a target or subject of annotation necessarily means a digital artifact – that is, a Web document, or more technically, an “information resource” [1]. The predicate or content of an annotation will be typically either a discourse about, or metadata about, the target. At times, an annotation can be as simple as a highlight. Multiple systems and technical approaches are used today for annotating information resources on the Web and for viewing the annotations in context.

Shared document annotation such as that available in Utopia (http://getutopia.com) [2] or Mendeley (http://www.mendeley.com) [3], are becoming increasingly popular in certain communities. There is also a growing interest in, and recognition of the importance of, using information extraction algorithms to perform semantic tagging of biomedical publications [4].

Biological textmining algorithm performance trials are organized annually by the Biocreative group (http://www.biocreative.org/) [5-11]. The National Center for Biomedical Ontology (NCBO) offers ontology-driven term extraction on biomedical text as a core service. Several academic groups are active developers of biomedical textmining applications.

Biocuration, or biomedical database resource annotation, is highly useful and prevalent in biomedical research, for example, the annotation of resources such as Wormbase [12] or Flybase [13-15] with terms from the Gene Ontology [16-18], or annotating a UniProtKB/Swiss-Prot entry to reflect newly discovered information about a new protein family in *A. thaliana *[19].

Every professional scientist does a very simple kind of annotation on a routine basis: citation of the bibliographic metadata of supporting evidence for the papers’ assertions. Publication of results in any peer-reviewed publication is impossible without it.

In whatever context it occurs, annotation is a key element of the process of doing science, as it supports the “virtual witnessing” process, characterized by Shapin as being fundamental to the scientific method since the first scientific journals and books began to be published in the 17^th^ century [20].

Web-based annotation is done today in multiple ways using various technical approaches and annotation representation formats. This is understandable considering the current transitional state of scientific publishing between print media and the Web. Furthermore, design decisions made in the early architecture of the Web itself (links embedded in the page) made development of unified tools and representations of Web annotation quite difficult before tooling and infrastructure of the semantic web became available.

Now is a good time to resolve this situation.

Our group has developed a useful software framework for performing, versioning, sharing and viewing Web annotations of a number of kinds, using an open representation model, the Annotation Ontology (AO) [21,22]. This framework – and in particular the Domeo user interface component – were developed in tandem with AO. A number of different alpha users provided concrete and practical use cases, which were incrementally added to the toolkit. We report on the Domeo user interface here. Future papers will describe other aspects of this work in detail, including the integration with the Apache UIMA framework through the open source Apache Clerezza-AO plugin, and the Annotation Framework Services.

## Results and discussion

### The Domeo Annotation Toolkit and the Annotation Ontology

The Domeo Annotation Toolkit (http://annotationframework.org) is a collection of software components that provides a rich set of features including

(i) semantic annotation of online HTML and XML documents;

(ii) automated, semi-automated and fully manual annotation protocols;

(iii) structured, semi-structured and unstructured annotation types;

(iv) full provenance of annotation and curation records;

(v) selective sharing of annotations;

(vi) serialization of annotations in RDF/XML using the Annotation Ontology; and

(vii) enhanced searching of annotations by leveraging semantic inference.

Domeo is currently in beta release.

As of this writing we support installations at the Massachusetts General Hospital; and at the University of California at San Diego (UCSD), as part of the NIH Neuroscience Information Framework (NIF, http://www.neuinfo.org/) and the NIH Blueprint for Neuroscience Research (http://neuroscienceblueprint.nih.gov/).

A full production version of Domeo will be included in the next release of the Neuroscience Information Framework.

Using Domeo, registered users can create unstructured, semi-structured and fully structured or semantic annotation on Web documents using this framework. It does not matter whether or not the documents themselves are under update control of the annotator. Annotation can be kept private, shared within selected groups, or made public and therefore available to the entire Web. These access control features enable personal as well as collaborative use of the tool.

The Domeo Toolkit was developed in parallel with the Annotation Ontology (AO), an OWL ontology providing a model for creating ‘stand-off’ annotation anchored to online resources such as documents, images and databases and their fragments [21,22]. AO provides a robust set of methods for linking online resources, for example text in scientific publications, to ontological elements, with full representation of the annotation provenance.

Through AO, existing domain ontologies and vocabularies – in OWL [23] or SKOS [24] - can be utilized, out of the box, for creating extremely rich stores of metadata on web resources. In the bio-medical field, subjects for ontological structuring include biological processes, molecular functions, anatomical and cellular structures, tissue and cell types, chemical compounds, and biological entities such as genes and proteins. However, AO is not limited to the bio-medical domain and can be easily used in other scientific and non-scientific contexts. In fact, AO is already used by other projects focusing on biodiversity [25] and social tagging [26,27].

AO, by linking new scientific content to computationally defined terms and entity descriptors, can help establish semantic interoperability across the diverse masses of specialist science embodied in digital media -- from journals, to wikis and blogs [28,29], to the growing world of web-based research “collaboratories” [30]. In biomedicine, semantic interoperability facilitates cross-species comparisons, pathway analysis, disease modeling, compact “mashups” for visualization, and the generation of new hypotheses through data integration and machine reasoning. Annotation can enrich the information content of web documents as well as contextualizing discussion about them. When annotation metadata take the form of controlled biomedical term sets, it can be used by software agents to enhance “strategic reading” [31,32].

While AO provides the model for encoding and sharing annotation in the convenient RDF (Resource Description Framework) format [33], it is still necessary to develop software applications allowing the users, in our case bio-medical scientists, to manually or semi-automatically create, share/publish, search and utilize annotation, and to manage algorithmically created annotation. As we strongly believe developing actual software is required to test the exchange model format against real use cases, we developed AO in parallel with AF.

In the following sections we describe some of the features of the current version of AF’s user interface web component, the Domeo annotation tool.

### The Domeo user interface

The Domeo user interface is an extensible web component enabling direct user-invoked annotation of online HTML/XHTML/XML documents. It was developed using a combination of Google Web Toolkit (GWT) and pure JavaScript. As the GWT code is eventually compiled into JavaScript, the final product consists of a JavaScript file to be imported by the hosting page. In order to work properly, the tool requires a series of services provided by the Domeo server component.

Domeo was designed to be part of the normal everyday workflow of scientists. It enables loading of online HTML documents that display as they would display when loaded directly in the browser. The task can be performed by copy and paste of the document URL in the Domeo address bar or, in a more efficient way, by using a Firefox plugin that adds the Domeo icon to the browser statusbar. With the plugin, when a user wishes to annotate the current web page, invoking the plugin via a single click, triggers re-loading of the page within the Domeo user interface.

### The Domeo server

A Domeo server can potentially be developed in any language or platform able to publish a web page accommodating the JavaScript of the Domeo web application. Our current Domeo services implementation runs currently relies on a Grails installation [34] and on a Java and Groovy [35] code base. MySQL (http://www.mysql.com/) supports the annotation repository. Communication between the server and the Javascript client is via JSON.

### Domeo web services

Domeo is designed to access [36], ontologies and other automated markup facilities via web service calls. We currently access vocabulary lookup, selection and entity recognition services through the NCBO Bioportal and Annotator web services hosted at Stanford University. These services are called when annotation vocabularies are selected, and when textmining is specified for a document or document section. Clearly automatic term recognition in biology is an evolving and highly competitive area, as can be seen by the fact that international competitions between various algorithms are regularly organized – see for example [37-41]. Our web services strategy is designed to enable Domeo to track and adapt to these changes in technology over time, and to differences amongst algorithms in fitness for particular purposes.

We also provide bibliographic reference lookup and identification through PubMed web services hosted at the National Center for Biomedical Information (NCBI). When a web document is loaded into Domeo, the software attempts to recognize its source. If the source is known and the source structure is available to Domeo, all the references are parsed to find PubMed and/or PMC identifiers, which are used to instantiate the complete bibliographic metadata for the article itself and its cited references as internal object references. This citation network is by default, shared as a “public” annotation in Domeo. If a bibliographic record already exists as a Domeo object reference, it will not be re-extracted. Over time, a Domeo installation will build up a large network of citations in the areas of interest being curated.

As the citation network extraction is run server-side, it is possible to pre-fetch substantial citation networks as required, although this is not a standard feature in our current beta deployment.

### Manual annotation with the Domeo user interface

Once the resource is loaded, the user may manually annotate the whole document, or sections of it, by selecting the desired portion of text and attaching a “topic”, representing an instance of one of several available annotation types. The simplest annotation types that can be created through Domeo are:

• highlight: A **highlight** is the process of marking a fragment of a resource with some - usually visual - mechanism.

• note: A **note** is defined as a brief record of facts, topics, or thoughts, written down as an aid to memory. When using the term 'note' we here refer only to free text notes.

• semantic tag: A **semantic tag** or, in AO terms, a **qualifier,** expresses a relationship between the target of the annotation and a well-defined semantic or structured entity consisting of a term of a controlled vocabulary or ontology identified by a URI.

The annotation process is enabled by a user interface widget that allows choosing and detailing the desired annotation type. In the case of qualifiers, the widget allows automatic term recognition (entity identification) on selected text, via an external web service accessed through a software connector. The current beta version connects to the NCBO (National Center for Biomedical Ontology) Annotator REST web service for terms search [42-44]. The NCBO search capability allows specifying some text and finding terms across multiple ontologies that contain it. The names, synonyms, and properties for a term are searched for matches to the entered text.

It is important to note that Domeo allows users to connect any search service and to customize the list of available vocabularies. By simply changing the set of vocabularies used for performing the annotation, it is possible to tackle any desired domain. It is even possible to re-use the BioPortal infrastructure for uploading and managing the desired ontologies and providing some of the web services necessary to Domeo to operate.

**Figure 1:**
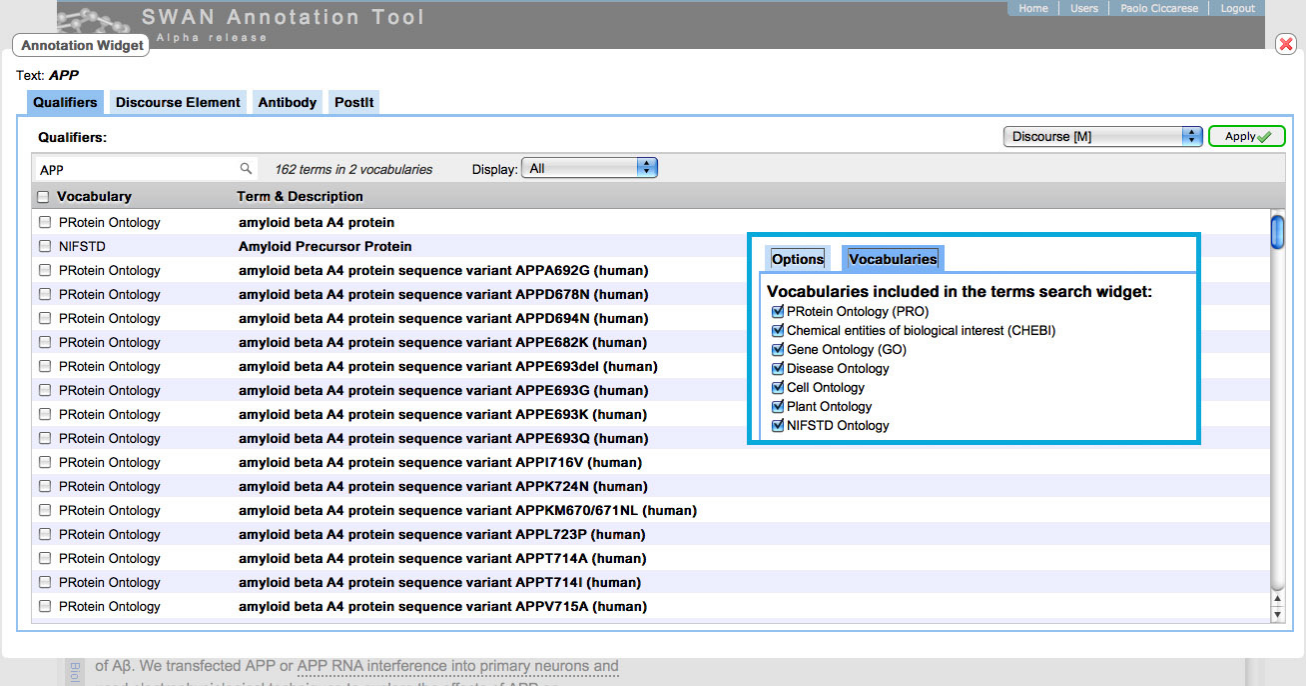
**NCBO BioPortal webservice search results**. Results of a terms search against the NCBO BioPortal through the BioPortal webservice. The results are currently displayed as a list and they include the terms belonging to the terminologies/ontologies enabled by the user’s preferences.

Domeo currently presents the search results in a linear list (Figure 1). However, motivated by users’ feedback, we are exploring alternative visualization that would allow users to browse the desired terminology or ontology.

**Figure 2:**
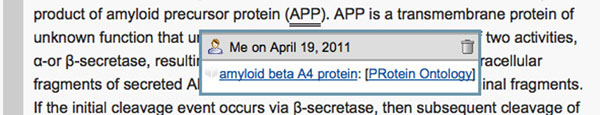
**Clicking on annotated text to inspect the associated annotation.** Users click on the annotated text to inspect the associated annotation items, in this example a semantic entity represented by a term from the PRotein Ontology. Some of the available provenance data are also displayed.

Once the annotation – in this case a qualifier – is created, the annotated span of text of the document is highlighted. Clicking on the span of text allows inspection of the annotation items associated with it, through a popup (Figure 2). Full annotation provenance - who created the annotation, when, with what tool... - is recorded, however only a summary is displayed by the popup.

The above annotation types are part of the standard distribution of Domeo. Additional types can be added by developing new software components or plugins. These define user interface components, semantic aspects of the new annotation topics, and connectors to external services when needed. Already developed plugins include features for modeling scientific discourse according to the model provided by the SWAN [45,46] ontology and features for modeling antibody usage. The latter has been developed in collaboration with the NIF (Neuroscience Information Framework) project and consists of annotating text with one of the antibody entries of http://antibodyregistry.org and, optionally, with the methods and species involved in the particular study reported in the document content.

### The SWAN ontology plugin

The SWAN plugin allows key assertions or claims in any paper to be recorded, along with their primary evidence in the literature, comments by the reader / reviewer / curator, and mappings to biomedical terminologies. Terminology mappings can be generated automatically – with user override – for any assertion. Bibliographic references are treated as structured semantic annotation, and are normalized against the National Library of Medicine’s article metadata using NCBI PubMed web services.

Annotation created by this plugin, as with all Domeo annotation, is independently retrievable, selectively shareable (for example with collaborators), and retains location-based contextualization in the original literature [47].

This plugin was initially designed to allow users to create SWAN content through Domeo. However we believe it will also be useful in the broader context of general scientific note-taking.

The SWAN project (Semantic Web Applications in Neuromedicine) developed a practical, common, semantically-structured, framework for scientific discourse, which can be applied to significant problems in Alzheimer Disease (AD) research and many other biomedical disorders. In the initial workflow, curators of AlzSWAN, the SWAN Alzheimer Database [48], used the SWAN Workbench to curate content which was then published via the SWAN Browser web application (http://hypothesis.alzforum.org). Output of SWAN’s curation process represented the article’s scientific discourse by means of formal discourse elements from the SWAN ontology: questions, hypotheses, claims,and evidence. For each discourse element the curator selected related publications (motivating or evidence), associated proteins and genes, plus a few other properties. The curator also connected certain discourse element for individual publications, with others already in the knowledge base.

It eventually became clear that because the curation software was removing the annotated discourse elements from their context in the original publications, they were also extracted from the normal scientific activity of reading, which focuses on articles, not databases. We wanted to restore this connection.

This limitation can be overcome with Domeo (Figure 4) using the SWAN ontology plugin. This module allows highlighting a section of a document and annotating it with a SWAN discourse element: Claim, Hypothesis, Question or Definition. It is possible to attach terms relevant to the discourse elements through a search against the NCBO BioPortal web service. Domeo also allows specification of evidence for claims, as represented by cited publications. Directly citing datasets in an archive such as Dataverse [49,50] is a new feature currently in development.

**Figure 3:**
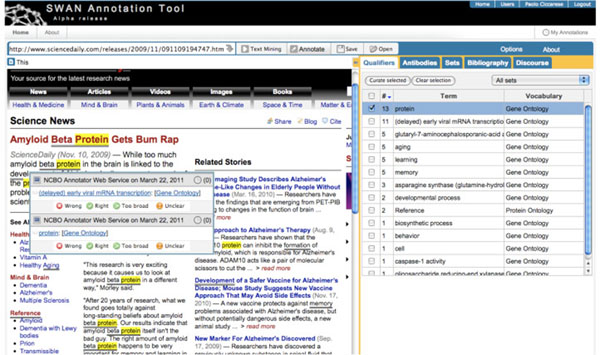
**Curation popup for user review and response to automatically generated annotation items**. The text-mining results are displayed on the document and the curation popup lets the user review and respond to automatically generated annotation items.

**Figure 4:**
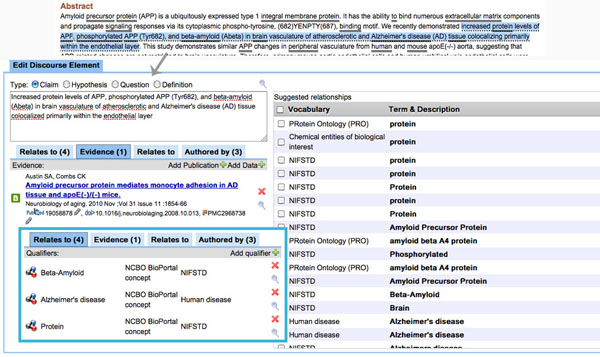
**Highlighted text fragment with associated discourse element creation panel.** Above, the highlight of the text fragment from the PubMed abstract at the URL http://www.ncbi.nlm.nih.gov/pubmed/19923279. Below, the discourse element creation panel. The left side of the interface is dedicated to the type and description of the statement together with all the related terms, publications, other statements in the knowledge base and authors. In this particular example, it is possible to see a supportive publication and the list of related ontological terms. The right section of the interface is used for suggestions and for searching for terms, evidence - publication and data, and other statements in the knowledge base.

**Figure 5:**
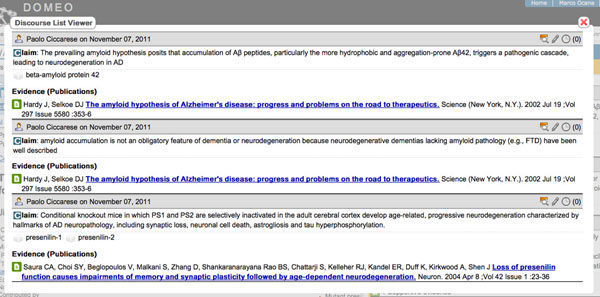
**View of the scientific discourse related to a publication**. Example of view of the scientific discourse related to a publication. Three claims have been encoded with their supportive evidence and related ontological terms.

Evidence, as modeled by the SWAN ontology, can be classified as supportive, inconsistent or relevant. In the current implementation, publications metadata are retrieved through a search in PubMed via NCBI web services. It is also possible to relate the new research statement with others already in the knowledge base once again through the relationships provided by the SWAN ontology: consistent, inconsistent and alternativeTo. The process is currently manual but we are planning to experiment with methods for retrieving automatically related statements in the knowledge base. Once the desired discourse elements have been created it is possible to see them summarized in the Domeo ‘discourse’ perspective (Figure 5).

### Semi-automatic annotation

In many cases, the efficiency of mass-scale manual annotation can be significantly augmented by annotation algorithms. DOMEO allows implementing the RECS (Run, Encode, Curate, Share) process. Using this process, it is possible to select and run external text mining services, encode the results in the AO format, display the results in the context of the annotated document (Figure 3) to enable the curation process. In the current version of the tool we integrated the NCBO Annotator web service. The NCBO Annotator is an ontology-based Web service that annotates public datasets with biomedical ontology concepts based on their textual metadata. It is possible, through the Domeo preferences panel, to specify which ontologies to consider when running the service. The current list of allowed ontologies for running the NCBO Annotator is the same list of ontologies allowed in the terms search through the NCBO bioportal mentioned in the previous sections.

Curation is a crucial aspect of scientific publication and therefore an important aspect for both our annotation ontology and our annotation tool. We enable curation for annotation generated by both humans and text mining services. In the case of automatic generated annotation, the tool allows curators to judge each annotation item (or set of annotation items) according to a configurable set of judgment categories. By default avalable categories are: “wrong”, “right”, “too broad”, “unclear” – where “unclear” means the curator is unable to understand or judge the result.

Every time a curator judges and responds to a result, s/he can also provide motivation that can be used later on for further evaluation. The users can also provide, through manual annotation, the list of entities that have been overlooked by the text mining algorithm. Eventually, the curated results and the terms suggestions can be exported and sent back to the text mining providers for measuring the performance of their tools or even for implementing incremental learning of their algorithms.

As several users may produce annotation on the same document, several users or curators may therefore curate the same results. The annotation tool enables both concurrent and collaborative annotation and curation processes.

### Provenance, access control and RDF sharing

**Figure 6:**
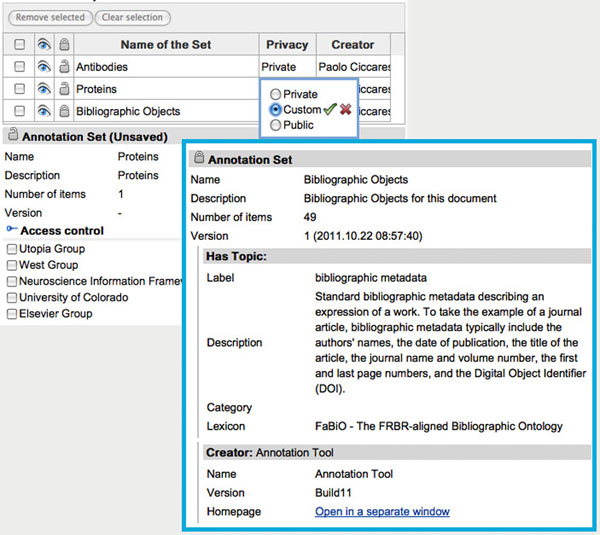
**Annotation Sets access control.** Annotation Sets access control.

In working with online scientific communities, we are particularly aware of the importance of provenance tracking for establishing trust and properly documenting evolution of the science. AO offers a rich set of properties for modeling provenance based on the Provenance Authoring and Versioning (PAV) ontology originally developed for the SWAN project [22,51]. Our annotation tool tracks all the provenance aspects transparently while the user performs the annotation process. For every piece of annotation and annotation curation, the tool records the originating user, date, and the specific version of any software or web service involved. DOMEO also implements another feature of AO: the Annotation Set, a mechanism for grouping annotation items. The notion of an Annotation Set was included in AO to assist in annotation organization. Sets can be used, for instance, to collect items of the same type – i.e. proteins or genes –, to show/hide multiple items, and to define the corresponding access policy. Using the annotation tool it is also possible to define, for each set, which users will be able to access the annotation items (Figure 6): only the creator (personal annotation), selected groups, or everybody (public annotation).

The annotation and curation items, together with all the provenance data, can be then serialized in RDF format according to the AO model. Serialization includes RDF representing aspects of the domain ontologies used in any annotation, as well as the AO RDF itself.

## Conclusions

The Domeo annotation tool is a web component developed using a combination of the Google Web Toolkit (GWT) and pure JavaScript. As the GWT code is eventually compiled into JavaScript, the final product consists in a JavaScript file to be imported by the hosting page. In order to work properly, Domeo needs a series of services that can be developed potentially in any language or platform able to eventually publish a page that accommodates the Domeo JavaScript component. After the Grails implementation, we are now in the process of starting the integration of Domeo with the Drupal open source Content Management System - using the PHP programming language. We are confident the same process could be replicated, in the near future, with other CMSs and programming languages.

Fifty alpha testers have been using Domeo in the past year. Users’ feedback has been important to fix bugs and drive user interface improvments. As today, the beta release for the NIF (Neuroscience Information Framework) is deployed within NIF and it is planned to be brought into production within one of the next releases of the NIF portal (probably version 4.2).

A detailed roadmap has been defined to further improve the features most important for text miners. Significant work has also been carried out to integrate Domeo services with the Apache UIMA framework, so that textminers using that architecture will be able to display and curate the results their text mining with our tool.

With the Domeo tool, the Domeo Annotation Framework in general, and the collaborations currently in place, we expect to be able to publish large quantities of high quality annotations on scientific documents in RDF AO format. The published annotation will include the content of the AlzSWAN knowledge base (http://hypothesis.alzforum.org) with the discourse elements – claims, hypotheses, and questions – linked to the correspondent text in original papers. We also note that annotation produced with our tool can be displayed on the corresponding PDF documents in the Utopia application [2,52] as Utopia can now consume AO RDF. We are currently working to with the Utopia group to enable the opposite workflow: producing annotation on a PDF of a scientific paper, and displaying it on the HTML version.

## Methods

Domeo was developed upon an initial set of requirements accumulated in developing curation-intensive biomedical knowledge bases and scientific online communities, including

• the AlzSWAN knowledge base (http://hypothesis.alzforum.org) [53], a customization of the Semantic Web Applications in Neuromedicine (SWAN) platform for hypothesis management in Alzheimer disease research;

• the Science Commons Antibodies Resource [54], an OWL model for formally representing antibodies and an associated collection of formally defined commercial antibodies;

• StemBook [55] (http://www.stembook.org), a web portal for the Stem Cell community collecting a comprehensive set of original review articles indexed by NLM; and

• PDOnline [56] (http://pdonlineresearch.org), a web portal for the Parkinson Disease researcher community, collecting several relevant resources including extensive online discussions by scientists.

Our approach was iterative and the application code was developed in tandem with the OWL representation of the annotation metadata in Annotation Ontology (AO). After completing an initial pilot and deploying some research code, we began taking on additional incremental use cases. These included:

• Annotation and curation of hypotheses in pharmaceutical drug discovery, based on requirements of a major international drug company;

• Annotation and curation of antibodies in literature, linked to the Neuroscience Information Framework (NIF) antibodies registry;

• Layering of multi-community annotation sets on documents;

• Curation, comparison and correction of annotations developed by automated textmining algorithms; and

• Creating scientific claims linked directly to evidence in the form of archived datasets.

For each of these use cases a corresponding community of users and user representative was identified. These were consulted extensively about detailed incremental requirements, user interface and fitness for purpose of the developed software. The NIF antibodies project now uses this toolkit on an ongoing basis. We have received several requests from biomedical textmining groups to use this software as well and are currently working to organize textmining users in a way that will make ongoing support as straightforward as possible.

## Availability and requirements

• Project name: Domeo

• Project home page: http://annotationframework.org

• Operating system(s):

○ Client: browser-based, platform independent

○ Server: Linux

• Programming language:

○ Client: Google Web Toolkit, Javascript

○ Server: JAVA and Groovy, using the Grails framework

• License: release under Apache 2.0 planned for January 2012

• Any restrictions to use by non-academics: compliance with Apache 2.0 license

• Current deployment status: beta release.

• Website: http://annotationframework.org

## List of abbreviations used

AF: Domeo Annotation Framework; AlzSWAN: Alzheimer Disease Hypothesis Knowledge Base; AO: Annotation Ontology; CMS: Content Mangement System; GWT: Google Web Toolkit; HTML: HyperText Markup Language; NCBO: National Center for Biomedical Ontology; NIF: Neuroscience Information Framework’; OWL: Web Ontology Language; PAV: Provenance, Authoring and Versioning Ontology; PDF: Portable Document Format; PHP: PHP: Hypertext Preprocessor; RDF: Resource Description Framework; RECS: (Run, Encode, Curate, Share); REST: Representational State Transfer; SKOS: Simple Knowledge Organization System; SWAN: Semantic Web Applications in Neuromedicine; URI: Uniform Resource Identifier; URL: Uniform Resource Locator; XHTML: eXtensible HyperText Markup Language; XML: eXtensible Markup Language.

## Competing interests

The authors of this paper declare that they have no competing interests.

## Authors’ contributions

PC is the principal author of the Annotation Ontology (AO), and the software architect and principal developer of the Domeo Annotation Toolkit. Dr. Ciccarese is the main contributor to this paper and produced the figures.

MO co-developed the server-side software and the first pilot of the Domeo annotation tool.

TC conceived of and provided overall guidance for development of the Domeo Annotation Toolkit and Annotation Ontology projects, wrote the background section of this paper, co-wrote other sections, did the overall editing, and is principal investigator of the project.
